# Complex Common and Internal Iliac or Aortoiliac Aneurysms and Current Approach: Individualised Open-Endovascular or Combined Procedures

**DOI:** 10.1155/2014/178610

**Published:** 2014-09-28

**Authors:** Thomas Kotsis, Louizos Alexander Louizos, Evangelos Pappas, Kassiani Theodoraki

**Affiliations:** ^1^Vascular Unit, 2nd Clinic of Surgery, School of Medicine, University of Athens, Aretaieion Hospital, Vas. Sophias 76, 115 28 Athens, Greece; ^2^1st Department of Anesthesiology, School of Medicine, University of Athens, Aretaieion Hospital, Vas. Sophias 76, 115 28 Athens, Greece

## Abstract

*Objective*. Bilateral internal iliac artery aneurysms constitute the utmost configuration of infrarenal aortoiliac disease. We detail characteristic aortoiliac disease patterns and reconstructive techniques we have used, along with a visualized decision-making chart and a short review of the literature. *Material and Methods*. A retrospective, observational study of twelve clinical cases of patients with aortoiliac disease are described. Two patients had a common iliac artery aneurysm and were managed by the application of inversed stent-grafts; another case was repaired by the insertion of a standard bifurcated stent-graft flared in the right common iliac artery and with an iliac branched device in the left iliac arterial axis. Open approach was used in 5 cases and in 4 cases a combination of aortouniliac stent-grafting with femoral-femoral bypass was applied. *Results*. Technical success was 100%. One endoleak type Ib in a flared iliac limb was observed and corrected by internal iliac embolism and use of an iliac limb stent-graft extension. We report 100% patency rate during 26.3 months of followup. *Conclusion*. Individualized techniques for the management of isolated iliac or aortoiliac aneurismal desease with special concern in maintaining internal iliac artery perfusion lead to elimination of perioperative complications and long-term durability and patency rates.

## 1. Introduction 

Iliac artery aneurysms (IAAs) participate in various aortoiliac aneurismal (AIA) patterns that frequently necessitate sophisticated reconstructions. An IAA represents a 50% arterial diameter increase, compared to normal; in absolute terms, common iliac arteries (CIAs) are considered aneurismal with diameter > 18.5 mm for men (normal: 1.23 ± 0.20 cm) and >15 mm for women (normal: 1.02 ± 0.19 cm); internal iliac artery's (IIA) diameter, in both genders, is 0.54 ± 0.15 cm [1, 2]. Occasionally, a CIAA looms as an abdominal aortic aneurysm (AAA) extension; coexistence of uni- or bilateral ectasia or concomitant IAA and AAA approximates 20–40% [3]. Isolated IAAs (prevalence 0.008–0.03% [4]) represent 2–11% of intra-abdominal aneurysms. Isolated CIAAs are found in 70% of isolated IAAs (20% are IIAAs), being frequently (30–50%) bilateral and 50–85% asymptomatic on diagnosis. The CIAAs expansion rate is 0.29 cm/y; as no rupture of a CIAA < 3.8 cm has been reported, elective repair of asymptomatic patients with CIAA ≥ 3.5 cm seems justified [5]. External iliac artery aneurysms (EIAAs) consist < 10% of isolated IAAs [6]; isolated IIAAs represent 0.4%–1.9% of arterial aneurysms and 0.04% of AIAs [1] with 38% rupture incidence at presentation and 58–80% mortality rate. Symptomatic IAAs mandate intervention, regardless of size. Key-point in aortoiliac diseases is the IIA circulation which is crucial for pelvic organs, sigmoid bed, and gluteal muscles. Salvage of both, previously patent, IIAs is welcome, while unilateral preservation is strongly suggested. Identification of vulnerable for hypogastric ischemia patients as long as clinical status weighting, dictate the approach type—open, combined or endovascular—regarding IIA circulation disturbance.

In this paper we present complex aortoiliac aneurismal patterns focusing on technical details and visualize solutions performed by us and reported in the literature.

## 2. Methods 

Twelve characteristic cases of patients with aortoiliac disease treated in our department (except from the last case) by the same surgeon, between 2004 and 2010, are presented (Table 1), having signed informed consent. This is a descriptive clinical study. The surgical route was chosen according to the medical status and the anatomic obstacles of each patient. Minimal anatomic requirements for EVAR repair were proximal neck length > 15 mm, proximal neck diameter 17–32 mm, suprarenal angulation ≤ 60°, proximal neck diameter increase ≤ 10°, distal fixation length > 10 mm, distal fixation diameter < 20 mm, and angle between the long axis of the aneurysm and the iliac axis < 60°. All patients received general endotracheal anesthesia and after intervention were followed up at least 24 hours at the intensive care unit (ICU). After discharge all patients were advised to have a duplex ultrasound examination of abdominal aorta and iliac arteries at 1, 3, and 12 months.

**Figure 1:**
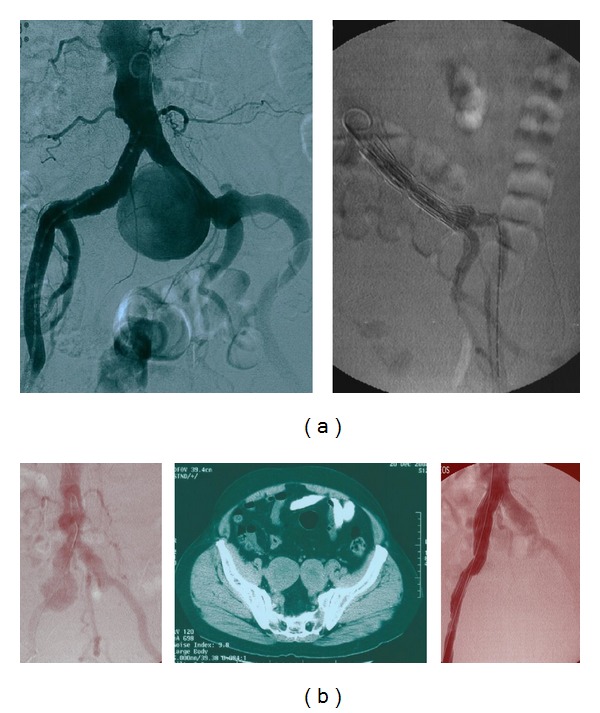
(a) Left common iliac artery aneurysm with large aneurysm's neck before and after implantation angiography; two concentric stents were inserted. (b) Right common iliac artery aneurysm with rich aortic collaterals and bilateral automatically thrombosed iliac artery aneurysms; an inverted stent-graft was implanted.

In the* 1st case* the patient had a large isolated left CIAA; one inverted stent-graft and a second extension (inlayed by usual deployment) stent-graft were used; the inverted graft method has been described in previous report [7] (Figure 1(a)).

In the* 2nd case*, the patient had an isolated right CIAA and bilateral IIAAs; both IIAAs were already thrombosed. Preoperative angiography revealed patency of the inferior mesenteric, a large middle sacral and many lumbar arteries; preoperative patient's erectile function was normal. An inverted stent-graft excluded the right CIAA [7] (Figure 1(b)); all other vessels remained intact.

**Figure 2:**
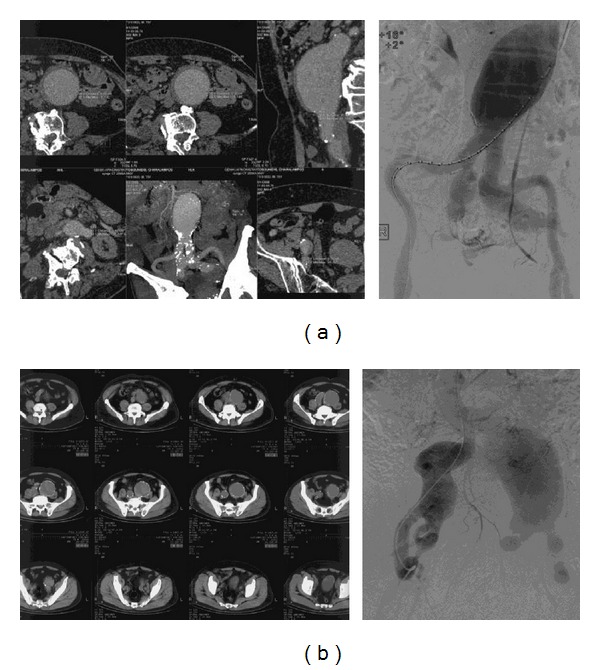
Two cases of complex aortoiliac aneurismal disease.

In the* 3rd case* of the patient with bilateral CIAAs and IIAAs (Figure 2(b)), following the proximal infrarenal anastomosis of an Y-graft, sigmoid cyanosis was evident; immediate end-to-end anastomosis of the right graft limb with the distal right IIA led to colon normalization; reperfusion of the legs followed.

**Figure 3:**
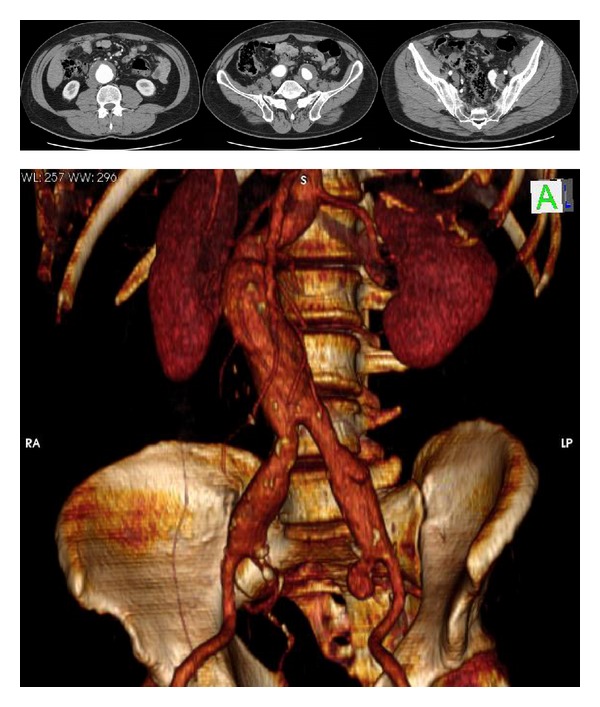
A 55-year-old patient with an AAA, bilateral common iliac, and left internal iliac artery aneurysms.

**Figure 4:**
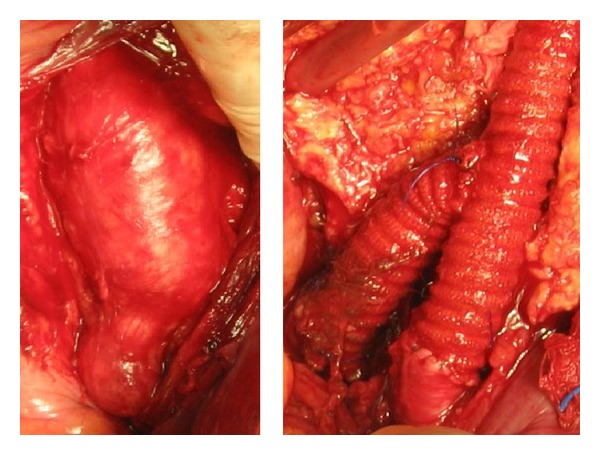
Intraoperative view of left common and internal iliac artery aneurysms and reconstruction.

In patients with concomitant AAA and IIAAs, we repair first the aneurysm of the planned for salvation IIA, following infrarenal aortoiliac exposure; prior to aortic cross-clamping, temporal flow exclusion of the aneurismal IIA and anastomosis with a 8 mm tube-graft is executed; then, standard aortoiliac or aortofemoral Y-graft is placed and the 8 mm tube-graft from the exposed IIA is implanted in the Y-graft limb, thus minimizing intestinal ischemia; we have used this method in two cases; in the patient with AAA and double right IIAAs (*4th case*), the 8 mm graft was anastomosed with distal right IIA and reimplanted to the Y-graft (Figure 2(a)) followed by endoaneurysmorrhaphy and ligation of the left IIA branches; in the* 5th case* both IIAs were preserved, commencing with the 8 mm graft interposition procedure for the left IIA (Figures 3 and 4).

**Figure 5:**
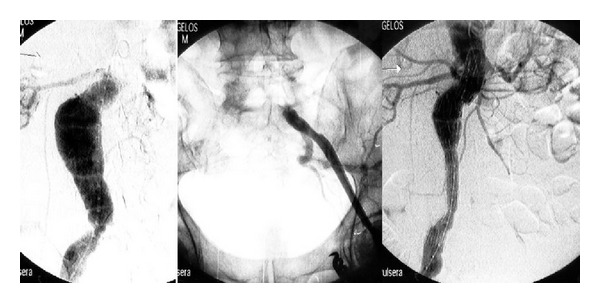
Concurrent AAA and left common iliac artery occlusion; an aortouniliac stent-graft insertion and a femoral-femoral bypass were performed.

A complex case (*6th case*) with AAA and left CIA occlusion was repaired with a right aortouniliac graft (Figure 5) followed by a femoral-femoral bypass (FFBP).

**Figure 6:**
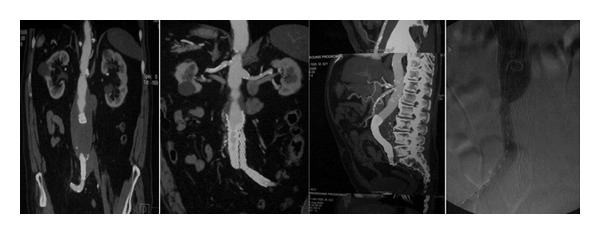
Migration and intense angulation of iliac graft limbs following EVAR, which necessitated the implantation of an aortouniliac stent-graft and a femoral-femoral bypass.

In the patient (*7th case*) with the migrated stent-graft following EVAR and highly angulated graft limbs, a right aortouniliac graft was deployed followed by FFBP; both IIAs were preserved (Figure 6).

**Figure 7:**
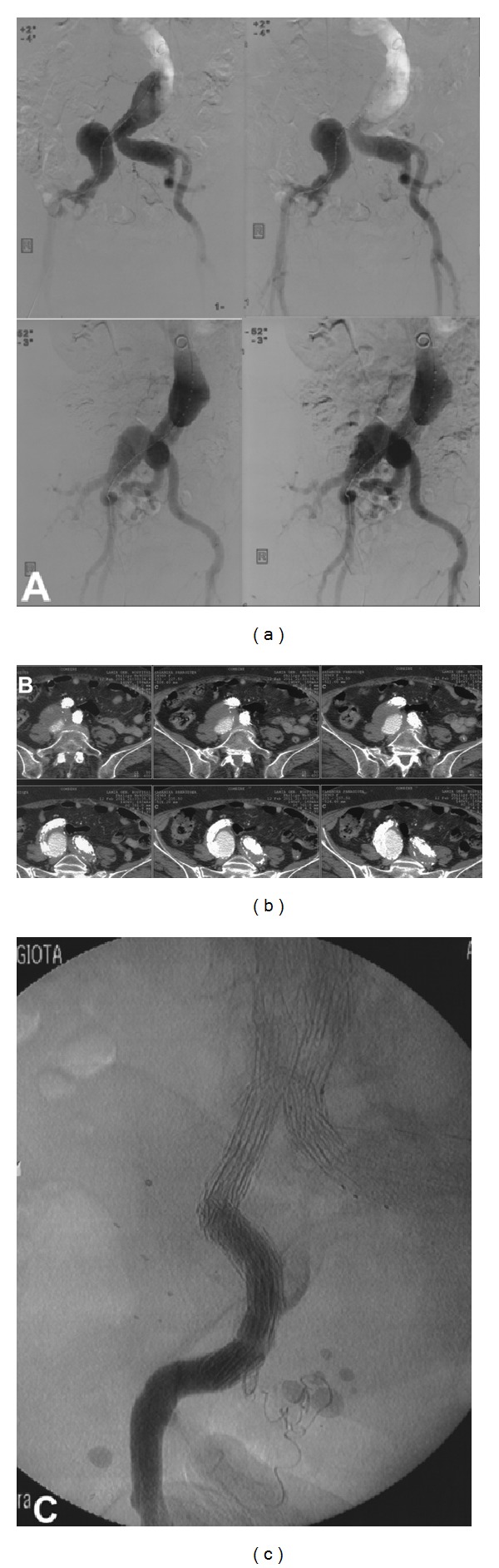
(a) A complex aortoiliac aneurismal pattern with bilateral CIAA and AAA. (b) Follow-up CT scan in one year showing endoleak type Ib in the flared right CIA and aneurysm sac expansion. (c) Completion angiography after right IIA embolization and extension stent grafts from right CIA to right EIA.

In the woman (*8th case*) with AAA and bilateral CIAAs, we used a bifurcated aortic stent-graft with a flared right leg and a left iliac branched device (IBD) (Figure 7(a)). The follow-up CT in one year showed expansion of the aneurysm of the flared right common iliac artery resulting in endoleak type Ib (Figure 7(b)). Embolism of the right internal iliac artery with coils followed by 2 stent-grafts extending from right common iliac to external iliac artery was used to repair this complication (Figure 7(c)).

**Figure 8:**
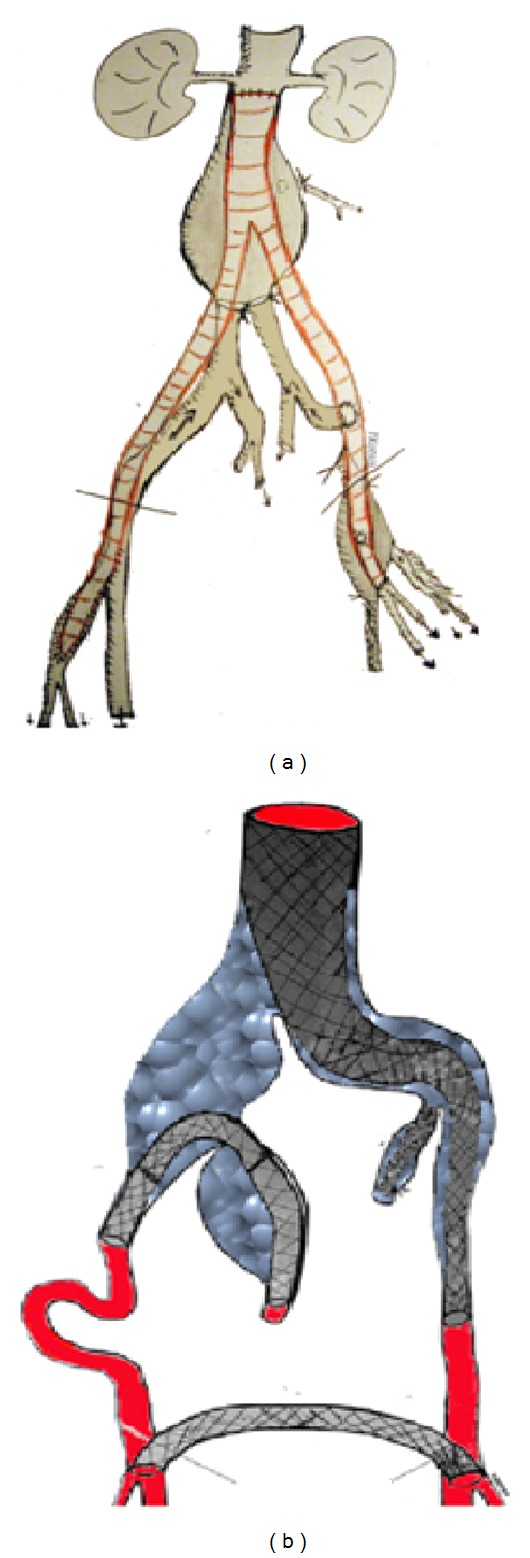
Two cases of complex open (a) and combined (b) reconstructions, respectively.

In the* 9th case* of the woman with the dissecting AAA and the right renal and right CIA malperfusion (Figure 8), right renal artery was fixated with perimetric sutures (2–8 hours) following deep-V shape aortotomy (12 hours) at the interrenal part; an aortobifemoral Y-graft was used; left IIA was perfused via antegrade flow from the left CFA; preoperative planning included hepatic-renal artery bypass.

In the* 10th case* of the patient with left iliac axis occlusion and concomitant AAA and right CIAA and stenotic EIA, a right aortouniliac stent-graft was implanted with an ipsilateral CIA to CFA bypass followed by a right iliac to left femoral bypass.

In the* 11th  case* with the concomitant common femoral artery aneurysm (CFAA), following distal aortic ligation at bifurcation, left IIA was antegrade reperfused via left CIA, as proximal left EIA was reimplanted to the aortobifemoral graft left limb (Figure 8(a)).

Finally, in the patient (*12th case*) with bilateral CIAAs and right IIAA, a combination of left aortouniliac stent-graft, left IIA embolization, reversed U-stenting of the right EIA to distal IIA, and a FFBP was performed; this case has been detailed in a previous report [8] (Figure 8(b)).

## 3. Results 

Perioperative and hospital mortality was nil. No patient suffered from renal impairment. Mean ICU stay was 2 days. During a mean follow-up (FU) of 26.3 months (min 9, max 56) one patient suffered a fatal cardiac arrest at 3rd postoperative year; patency rate of all grafts was 100%. In one patient, an incomplete right leg ischemia was present on 1st postoperative day, which was assisted by a femoral-femoral bypass; in another patient, a CFAA was noticed in the FU CT almost 3 years postoperatively, and another presented postoperative uncomplicated left pelvic pain and an abdominal hernia. One endoleak type 1b was observed due to fixation loss of the flared stent-graft's right limb (*8th case*). There was no further secondary operation for these patients.

## 4. Discussion

Systemic cardiovascular disease along with other comorbidities is frequently encountered in patients with aortoiliac aneurysms mandating a precise preoperative patient assessment in order to offer the “definite” repair with the least risk [9, 10].

Open procedures are commonly considered as more durable, though for fit patients like younger patients. On the other hand, elderly patients who may bear a major restrictive cardiopulmonary disease may be managed with endovascular or combined methods with occasionally questioned long-term endurance. Correction of iliac or aortoiliac aneurismal with or without occlusive disease focuses on the salvation of both or at least one IIA. Regardless of the chosen tactic, IIA perfusion is strongly suggested, especially when compromised collateralization due to prior surgery or atherosclerosis is suspected.

Ischemic damage of organs perfused by distal IIA branches is irreversible and IIA occlusion complications include sigmoid damage, spinal cord ischemia, hip and/or buttock claudication or necrosis [11], sexual erection dysfunction, or testicular infraction. Patients with rich femoral or lumbar collaterals may tolerate IIA occlusion but this cannot be universally anticipated [12, 13], and unfortunately when EVAR is applied, stent-grafts cover lumbar and inferior mesenteric artery orifices. Unilateral IIA open ligation or endovascular embolization to facilitate surgical graft or endovascular stent-graft implantation, respectively, is also questioned; right scheduled IIA occlusion is preferred due to direct left IIA-sigmoid bed collateralization; arteriographic IIA critical stenosis > 70%, ipsilateral CFA collateral loss, or perfusion absence in three or more ipsilateral IIA branches are critical. In a review of 301 patients who underwent uni- or bilateral IIA occlusion [14], ischemic complications followed; two-step bilateral IIA occlusion for collateralization offers no clinical benefit [14] whilst unilateral IIA occlusion (e.g., for prevention of endoleak in EVAR) is debated; hypogastric embolization results in worse pelvic ischaemia versus simple stent-grafting. In a study of 147 patients requiring IIA uni- or bilateral occlusion [15], buttock claudication incidence was higher (6-month FU) in patients with IIA embolization (42%) versus IIA coverage (8%) but, impressively, endoleak incidence remained the same; reduced complications also arise from proximal IIA compared to distal embolization [14]. In a meta-analysis of 634 patients [16] buttock claudication occurred in 28% (178 of 634 patients): in 31% (99 of 322) of unilateral and 35% (34 of 98) of bilateral embolizations; new erectile dysfunction occurred in 17% (16 of 97) of unilateral and 24% (9 of 38) of bilateral embolizations. Despite life-saving IIA ligation/embolization [11] in emergencies [17, 18], complications do occur; thus an IIA salvation plan is required.

Open repair employs an aortoiliac or aortofemoral (or combinations) Y-graft with distal anastomoses or reimplantation of healthy iliac arteries' segments (Table 2); however, bilateral or unilateral IIAAs presence is challenging due to deep pelvic location and the frequent previous AAA repair; relatively higher operative mortality (~10%) [19] than AAA repair is reported, although series with isolated IAAs open repair report good midterm results [6, 20, 21].

The IIA reimplantation to the graft is commonly used; Millite et al. [22] reported good mid-term procedural patency and safety of IIA bilateral in 18 and unilateral bypass in 8 patients with a polyester prosthesis. Open procedure is effective in symptomatic IAAs compressing adjacent structures [23].

The exception for the rule, concerning open repair, is the isolated CIIA, like in the first two cases of patients we present, where anatomy (existence of iliac proximal and distal neck) allowed minimal invasive pure endovascular repair; even if stent-grafting fails, secondary operations in iliacs are less complicated and mostly can be reaccomplished by catheters. In the first of the aforementioned cases, the stent-to-stent technique provided the necessary columnar support and solitary stent-graft collapse into the large aneurysm was obviated (Figure 1(a)). By implanting an inversed stent-graft [7] in the patient with the right CIA (Table 1,* 2nd case*, Figure 1(b)) and not an aortic Y-stent-graft, type II endoleaks were avoided and patient's erectile function was preserved.

In open repair, sigmoid surveillance is needed [24, 25]. In the patient with aneurysmosis, (Table 1,* 4th case*, Figure 2(b)), sigmoid normalized following IIA reperfusion. In complex aortoiliac aneurismal disease with aneurismal IIAs, we repair first the aneurysm of the selected for salvation IIA (the left is preferred or the one with the smaller aneurysm) thus minimizing ischemia time; as far as we know this is the first report of this strategy. In another case with a concomitant CFAA, where dissection above inguinal ligament was necessary, reperfusion of the ipsilateral IIA was succeeded via proximal left EIA reimplantation to Y-graft limb (Figure 8(a)).

When IIA preservation through median laparotomy and endotracheal anaesthesia is unsafe, pure endovascular repair is attractive for high risk patients; however, EVAR application with standard stent-grafts is limited due to anatomic proximal aortic morphology or due to concomitant CIAAs or IIAAs which lead to unavoidable IIA occlusion due to lack of favorable distal landing zone; clinical EUROSTAR data also showed that EVAR in aortoiliac aneurysms using conventional stent-grafts was associated with higher type I endoleak incidence, secondary interventions, and delayed rupture [3]. Combinations of endovascular and minimal open procedures for IIA reperfusion via retroperitoneal direct IIA to EIA or via FFBP may provide a more stable/uncomplicated operation; combined procedures can be accomplished through epidural, dorsal, or local (in FFBP cases) anesthesia. Aortouniiliac and subsequent FFBP is an effective technique especially in case of rupture [26] and in high risk patients with one iliac axis occlusion; we have used this approach in 4 cases; in two of them the contralateral iliac axis (Figure 5) was occluded so in one case a FFBP and in the other a retroperitoneal right iliac axis repair and an iliac-femoral right to left bypass followed. The patient with the bifurcated stent-graft from another department which five years later migrated is a characteristic case of conversion due to migration; aortouniliac stent-grafting provides the necessary columnar strength in highly angulated graft limbs (Figure 6).

Parodi and Ferreira [27] suggested surgical implantation of IIA to distal EIA, through a lower abdominal incision, in order to extent the EIA distal landing zone without compromising IIA flow. Bergamini et al. [28] proposed retrograde endografting from EIA to IIA followed by contralateral hypogastric coil embolization; then, an aortouniliac stent-graft extending to the contralateral EIA is implanted, followed by a FFBP. Similar combined techniques have been performed by Derom et al. [29] and Clarke et al. [30] while we have used the same technique for bilateral CIAAs and for the first time in an aneurismal IIA [8]; useful tips are the long introducer to overcome iliac angulation and balloon infusion to facilitate IIA catheter advancement [8]. Leon et al., in order to maintain left IIA perfusion in a patient with a prior bifurcated prosthesis for AAA (with inferior mesenteric artery and right IIA ligated), deployed two overlapping covered stent-grafts, extending from the proximal aneurismal left CIA to the left IIA along with FFBP [7]. This technique is also reported by Woo et al. in a patient with paranastomotic post-AAA repair CIA aneurysms who underwent CIAAs exclusion by an aortouniliac endograft along with endovascular EIA-IIA and FFBP [31]. Delle et al. [32] proposed unilateral IIA embolization followed by endograft main body deployment in the ipsilateral EIA; via branchial artery a covered-stent deployed into the cannulated contralateral IIA excluded the aneurysm sac, followed by ligation of the nonperfused EIA and FFBP.

Patients with lower life expectancy, comorbidities, and hostile abdomen, and as stated those with isolated CIAAs, are candidates for pure endovascular repair. Stent-grafting anatomical suitability is based on preoperative CT studies; excessive iliac tortuosity along with circumferential vessel wall calcification and significant arterial occlusive disease through which endoluminal access is planned consist hostile anatomy for endovascular intervention [33, 34]. In case of stenotic CIAs or EIAs, angioplasty may facilitate luminal access; in patients where distal EIA diameter prohibits endovascular access, a stent-graft may be implanted via retroperitoneal access through a sutured tube graft (conduit) to EIA or CIA. Aneurismal extension to the IIAs' orifices may lead to IIA occlusion.

In case of insufficient CIA distal landing zone for aortoiliac aneurysms, bell-bottom technique with a flared-cuff to the unfavorable landing zone has been proposed [35]; this cuff anchors the device in the CIA, thus preserving IIA flow, and is recommended to patients with CIA maximum diameter of 28 mm, since CIA diameter > 30 mm increases the rupture risk. Use of flared iliac limbs in aortoiliac repairs results in 10% iliac reintervention and 7% type Ib endoleak rate at 30-month mean FU [36]. We have used the bottom-bell technique for the right CIAA in the woman with aneurysmosis which eventually failed. The flared iliac limb is contraindicated in patients with aneurysms involving iliac bifurcation, where IIA embolization or open surgery should be planned accordingly. Frigatti et al. [37] proposed a chimney-double-barrel technique including left IIA embolization, deployment of two overlapped endografts delivered from the right IIA to the distal aorta, and CIAA exclusion using iliac contralateral leg and extension endograft from distal aorta to both EIAs.

Isolated IIAAs pose a demanding challenge for endovascular repair. Parsons et al. [38] reported 86% 3-year patency rate for PTFE-stents treating IIAAs with 12% procedural complication rate and no CT increase in aneurysm's diameter (mean FU: 24 months). Boules et al. [39] reported FU of 45 patients with endovascular repair of 61 isolated IAAAs, with 96% 2-year primary patency and 88% freedom of secondary interventions.

Recently, Iliac Branched Devices (IBDs) have been proposed to partially solve IIA orifice engagement; the IBD was used for the preservation of the left IIA in the case of the woman with aortoiliac aneurysmosis (Table 1,* 8th case*, Figure 7). Historically, Greenberg used fenestrated devices in 21 CIAAs cases (mean CIAA size: 3.8 cm) and bilateral CIAAs in 18 of 21 patients [40]; failure to access IIAs occurred in 3 cases whilst 2 late hypogastric branch thromboses occurred; however these initial results were promising enough to lead to IBDs commercialization. Indications for IBD are in conjunction with EVAR for AAA with concomitant CIAAs or as an alternative to a flared cuff along with an EVAR graft, like in our case; it is also beneficial in case of isolated CIAAs with safe proximal neck. The IIA diameter must be <11 mm with IIA length > 10 mm and CIA diameter > 20 mm and length > 50 mm in order to receive the IBD [41]. Patients with IIAAs have also been treated with IBDs [42]; stenosis of the IIA is prohibitive for IBD.

Theoretically, IBDs may be used for bilateral CIAAs for preserving both IIAs. Preoperative high resolution CT-angiography study is required along with minimal IIA tortuosity and long landing zone. In case of IIAA and inadequate length of distal IIA landing zone for the IBD, an IIA branch may serve as IBD's landing zone with deliberate other branch occlusion. In a meta-analysis from Naik [42] nine reported series for IBDs, embodying 196 patients, were studied; there were 24 IBD occlusions in all series (FU: 6 months to 5 years). Technical factors predisposing to IBD occlusion are reported to be a sharp aortic bifurcation, iliac tortuosity and/or calcification, presence of intraluminal CIA thrombus, severe EIA kinking and IIA ostium stenosis, IIA atherosclerosis, and IIA aneurysm. Low endoleak rate was reported, with 1 type I and 2 type III endoleaks; however 5 EIA occlusions occurred; extension of the endograft into the EIA rises the iliac limb occlusion risk. These reported mid-term results are encouraging for this emerging technology but the relatively high IIA limb occlusion rate along with the extra cost of IBDs requires cost efficiency criteria and risk factors' formalization in order to identify those high risk patients who will exclusively need IBDs.

## 5. Conclusion 

In this study we aimed at providing technical considerations concerning aortoiliac aneurysms' management reviewing most of current options emphasizing on the individualization of treatment. By presenting these characteristic cases, our intention was to enlighten the diverse aspects of aortoiliac repair underlining that they are combined in a complimentary and not antagonizing association; we presume that patients' best outcome arises rather from optimum preoperative matching and not by adherence to a particular method.

Improper strategy concerning IIA reperfusion may promptly result in hindgut ischemia [24, 25] or in late pelvic or gluteal discomfort; nonabsolute repair concerning EIA reperfusion could result in early or late acute leg ischemia, a situation of major suffering. When aortic and CIA aneurysms coexist, open repair or pure endovascular repair by using a bifurcated stent-graft may be performed. For isolated CIAAs with good iliac proximal and distal neck, catheter techniques are advantageous. Bilateral isolated CIAAs with insufficient distal landing zone demand uni- or bilateral IIA reperfusion by open or a combined repair likely elaborating extraperitoneal IIA reperfusion or either by one or two IBDs (likely supported by a bifurcated aortic stent-graft), if anatomy permits. When uni- or bilateral IIAAs exist, favorable distal IIA healthy segment has to be investigated for covered-stent or IBD safe landing; open or extraperitoneal approach in such cases is demanding. Comorbidities and surgical experience determine the type of repair. Open surgery is a long established method [19]; for high risk patients pure endovascular repair, within anatomic limitations, can be exploited while combined techniques constitute a realistic option. It should be emphasized that there is no method that fits to all cases; individualization is the best therapeutic principle.

## Figures and Tables

**Table 1:** Descriptive table of all of our reported cases.

		Aneurysm					Occlusion				Operations				
Cases	Gender/age/ASA	Aortic	Common iliac		Internal iliac		Common iliac		Internal iliac		Type		Complications	FU/months	ICU/days
			RT	LT	RT	LT	RT	LT	RT	LT					
(1) Figure 1(a)	m/78/II	−	−	+	−	−	−	−	−	−	E	2 concentric (proximal inverted) stent-grafts	−	9	1
(2) Figure 1(b)	m/74/II	−	+	−	+	+	−	−	+	+	E	1 inverted stent-graft	−	22	1
(3) Figure 2(a)	m/77/II	−	+	+	+	+	−	−	−	−	OP	Y-graft aortoiliac right limb to IIA-CFA Left IIA ligation Femoral-femoral bypass	Intraoperative sigmoid ischemia Postoperative incomplete right leg ischemia	56	3
(4) Figure 2(b)	m/75/II	+	−	−	++	+	−	−	−	−	OP	8 mm right IIA graft Left IIA ligated Y-graft aortoiliac	Left pelvic pain Abdominal hernia	27	2
(5) Figures 3 and 4	m/55/I	+	+	+	−	+	−	−	−	−	OP	8 mm left IIA graft Aortoiliac Y-graft	−	18	2
(6) Figure 5	m/73/III	+	−	−	−	−	−	+	−	−	C	Aortouniliac right Femoral-femoral bypass	−	34	1
(7) Figure 6	m/80/*Ι* *Ι* *Ι*	+ Prior EVAR	−	−	−		Graft angulation	Graft angulation	−	−	C	Aortouniliac right Femoral-femoral bypass	−	24	1
(8) Figure 7	f/70/III	+	+	+	−	−	−	−	−	−	E	IBD left Bifurcated stent-graft-flare right	Endoleak type Ib of the flared CIA in 1 year FU	23	3
(9)	f/64/III	+ Type B dissection	−	−	−	−	+	−	−	−	OP	Right renal artery fixation Aortobifemoral Y-graft	−	25	3
(10)	m/72/III	+	+	−	−	−	−	+	−	−	C	Aortouniliac right Ipsilateral CIA-CFA bypass Iliaco-femoral bypass (right to left)	−	14	3
(11) Figure 8(a)	m/73/II	+	−	−	−	−	−	−	−	−	OP	Left femoral artery aneurysm aortobifemoral Y-graft Left EIA to left graft limb anastomosis	−	34	3
(12) Figure 8(b)	m/78/IV	−	+	+	+	−	−	−	−	−	C	Right EIA to right IIA covered stent (reverse-U stent) Left IIA embolism Aortouniliac left Femoral-femoral bypass	Left CFAA	30 Cardiac arrest	1

Type = type of operation (OP = open, E = endovascular, C = combined), ++ = double aneurysm, CFAA = common femoral artery aneurysm, IIA = internal iliac artery, EIA = external iliac artery.

**(a):** 

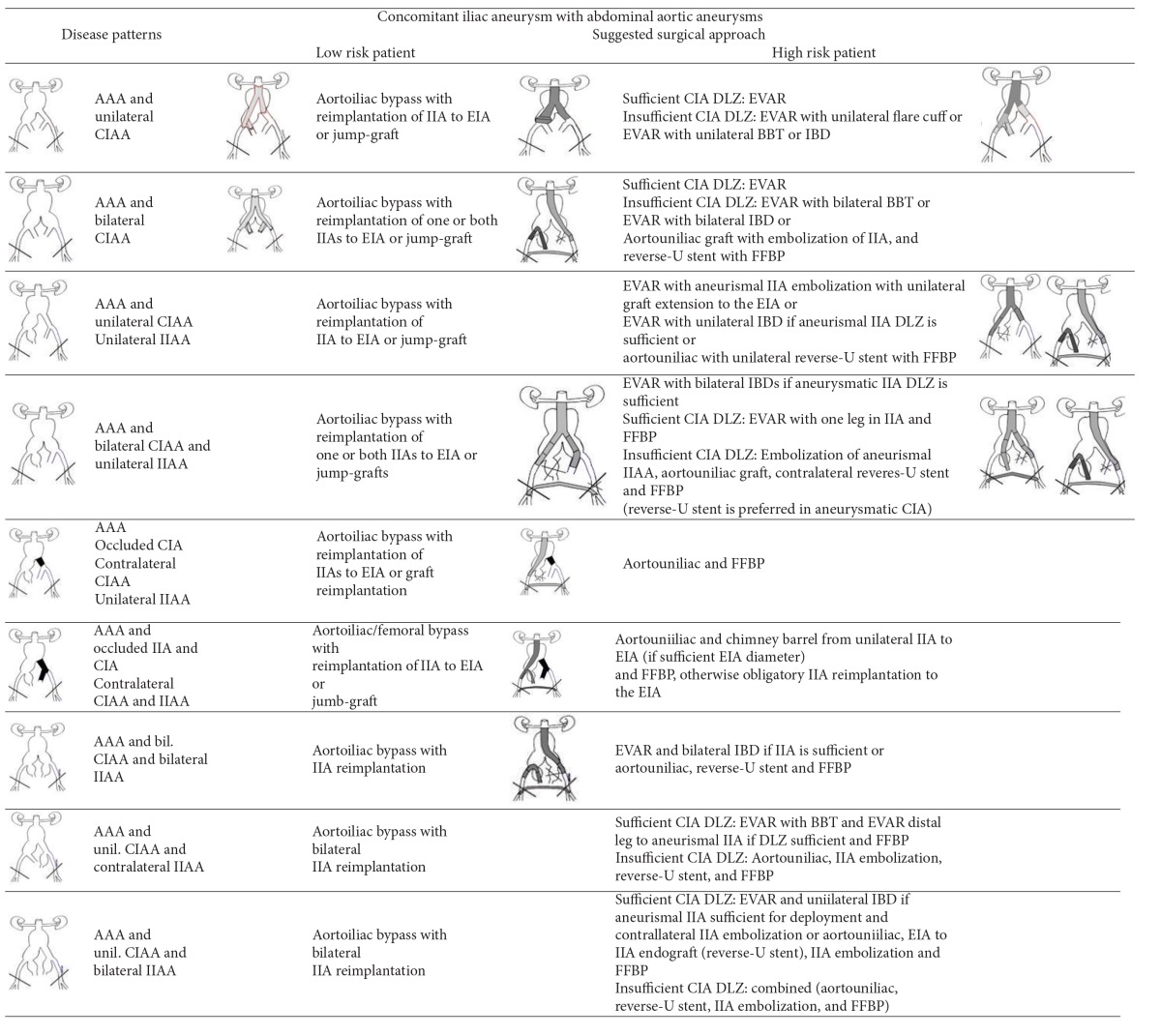

(i) In this table we consider that there is enough proximal subrenal aortic landing zone. In juxtarenal or thoracoabdominal aneurysms we consider the use of fenestrated stents or hybrid surgical interventions.

(ii) The possibility for combined endovascular and IIA to EIA implantation or jump graft through retroperitoneal access may be applied in all cases of aortoiliac aneurismal disease.

**(b):** 

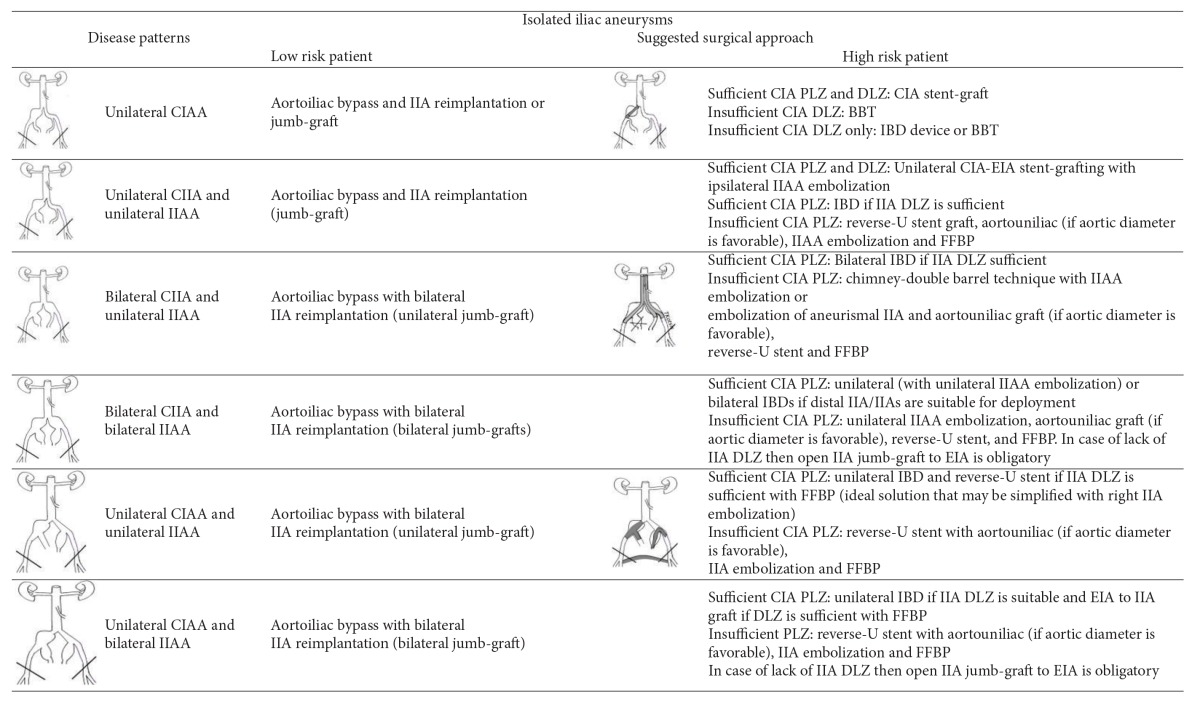

(i) External Iliac artery aneurysm patterns are not reported in this table because of their extremely low frequency.

(ii) FFBP = Femoral-femoral bypass, BBT = Bell Bottom Technique, IBD = branched iliac device, DLZ = distal landing zone, PLZ = proximal landing zone.

CIA = common iliac artery, EIA = external iliac artery, IIA = internal iliac artery.

## References

[1] Johnston KW, Rutherford RB, Tilson MD, Shah DM, Hollier L, Stanley JC (1991). Suggested standards for reporting on arterial aneurysms. Subcommittee on Reporting Standards for Arterial Aneurysms, Ad Hoc Committee on Reporting Standards, Society for Vascular Surgery and North American Chapter, International Society for Cardiovascular Surgery. *Journal of Vascular Surgery*.

[2] Horejs D, Gilbert PM, Burstein S, Vogelzang RL (1988). Normal aortoiliac diameters by CT. *Journal of Computer Assisted Tomography*.

[3] Hobo R, Sybrandy JEM, Harris PL, Buth J (2008). Endovascular repair of abdominal aortic aneurysms with concomitant common iliac artery aneurysm: outcome analysis of the EUROSTAR experience. *Journal of Endovascular Therapy*.

[4] Brunkwall J, Hauksson H, Bengtsson H, Bergqvist D, Takolander R, Bergentz S-E (1989). Solitary aneurysms of the iliac artery system: an estimate of their frequency of occurrence. *Journal of Vascular Surgery*.

[5] Huang Y, Gloviczki P, Duncan AA (2008). Common iliac artery aneurysm: expansion rate and results of open surgical and endovascular repair. *Journal of Vascular Surgery*.

[6] Krupski WC, Selzman CH, Floridia R, Strecker PK, Nehler MR, Whitehill TA (1998). Contemporary management of isolated iliac aneurysms. *Journal of Vascular Surgery*.

[7] Leon LR, Mills JL, Psalms SB, Goshima K, Duong ST, Ukatu C (2007). A novel hybrid approach to the treatment of common iliac aneurysms: antegrade endovascular hypogastric stent grafting and femorofemoral bypass grafting. *Journal of Vascular Surgery*.

[8] Kotsis T, Tsanis A, Sfyroeras G, Lioupis C, Moulakakis K, Georgakis P (2006). Endovascular exclusion of symptomatic bilateral common iliac artery aneurysms with preservation of an aneurysmal internal iliac artery via a reverse-U stent-graft. *Journal of Endovascular Therapy*.

[9] Bauriedel G, Skowasch D, Lüderitz B (2007). Perioperative cardiac risk stratification for noncardiac surgery. *Deutsches Arzteblatt*.

[10] Patterson BO, Holt PJE, Hinchliffe R, Loftus IM, Thompson MM (2008). Predicting risk in elective abdominal aortic aneurysm repair: a systematic
review of current evidence. *European Journal of Vascular and Endovascular Surgery*.

[11] Suzuki T, Kataoka Y, Minehara H (2008). Transcatheter arterial embolization for pelvic fractures may potentially cause a triad of sequela: gluteal necrosis, rectal necrosis, and lower limb paresis. *The Journal of Trauma*.

[12] Yano OJ, Morrissey N, Eisen L (2001). Intentional internal iliac artery occlusion to facilitate endovascular repair of aortoiliac aneurysms. *Journal of Vascular Surgery*.

[13] Mayer D, Pfammatter T, Rancic Z (2009). 10 years of emergency endovascular aneurysm repair for ruptured abdominal aortoiliac aneurysms: lessons learned. *Annals of Surgery*.

[14] Bratby MJ, Munneke GM, Belli A-M (2008). How safe is bilateral internal iliac artery embolization prior to EVAR?. *CardioVascular and Interventional Radiology*.

[15] Farahmand P, Becquemin JP, Desgranges P, Allaire E, Marzelle J, Roudot-Thoraval F (2008). Is hypogastric artery embolization during Endovascular Aortoiliac Aneurysm Repair (EVAR) innocuous and useful?. *European Journal of Vascular and Endovascular Surgery*.

[16] Rayt HS, Bown MJ, Lambert KV (2008). Buttock claudication and erectile dysfunction after internal iliac artery embolization in patients prior to endovascular aortic aneurysm repair. *CardioVascular and Interventional Radiology*.

[17] Dubose J, Inaba K, Barmparas G (2010). Bilateral internal iliac artery ligation as a damage control approach in massive retroperitoneal bleeding afterpelvic fracture. *Journal of Trauma—Injury, Infection and Critical Care*.

[18] Sidhu HK, Prasad G, Jain V, Kalra J, Gupta V, Khandelwal N (2010). Pelvic artery embolization in the management of obstetric hemorrhage. *Acta Obstetricia et Gynecologica Scandinavica*.

[19] Richardson JW, Greenfield LJ (1988). Natural history and management of iliac aneurysms. *Journal of Vascular Surgery*.

[20] Dorigo W, Pulli R, Troisi N (2008). The treatment of isolated iliac artery aneurysm in patients with non-aneurysmal aorta. *European Journal of Vascular and Endovascular Surgery*.

[21] Ferreira J, Canedoa A, Branda D (2010). Isolated iliac artery aneurysms: six-year experience. *Interactive Cardiovascular and Thoracic Surgery*.

[22] Milite D, Campanile F, Tosato F, Pilon F, Zaramella M (2010). Hypogastric artery bypass in open repair of abdominal aortoiliac aneurysm: a safe procedure. *Interactive Cardiovascular and Thoracic Surgery*.

[23] Sugimoto A, Haga M, Motohashi S, Takahashi Y, Kanazawa H, Nakazawa S (2011). A case of rectal obstruction caused by bilateral internal iliac artery aneurysms. *Annals of Vascular Surgery*.

[24] Mitchell KM, Valentine RJ (2002). Inferior mesenteric artery reimplantation does not guarantee colon viability in aortic surgery. *Journal of the American College of Surgeons*.

[25] Kotsis T (2002). Inferior mesenteric artery reimplantation. *Journal of the American College of Surgeons*.

[26] Ohki T, Veith FJ (2000). Endovascular grafts and other image-guided catheter-based adjuncts to improve the treatment of ruptured aortoiliac aneurysms. *Annals of Surgery*.

[27] Parodi JC, Ferreira M (1999). Relocation of the iliac artery bifurcation to facilitate endoluminal treatment of abdominal aortic aneurysms. *Journal of Endovascular Surgery*.

[28] Bergamini TM, Rachel ES, Kinney EV, Jung MT, Kaebnick HW, Mitchell RA (2002). External iliac artery-to-internal iliac artery endograft: a novel approach to preserve pelvic inflow in aortoiliac stent grafting. *Journal of Vascular Surgery*.

[29] Derom A, Vermassen F, Ongena K (2000). Endograft exclusion of residual common iliac artery aneurysms. *Journal of Endovascular Therapy*.

[30] Clarke MJ, Pimpalwar S, Wyatt MG, Rose JDG (2001). Endovascular exclusion of bilateral common iliac artery aneurysms with preservation of internal iliac artery perfusion. *European Journal of Vascular and Endovascular Surgery*.

[31] Woo EY, Lombardi JV, Carpenter JP (2004). Endovascular external-to-internal iliac bypass as an adjunct to endovascular aneurysm repair for patients with extensive common iliac artery aneurysmal disease. *Journal of Vascular Surgery*.

[32] Delle M, Lönn L, Wingren U (2005). Preserved pelvic circulation after stent-graft treatment of complex aortoiliac artery aneurysms: a new approach. *Journal of Endovascular Therapy*.

[33] Leurs LJ, Kievit J, Dagnelie PC, Nelemans PJ, Buth J (2006). Influence of infrarenal neck length on outcome of endovascular abdominal aortic aneurysm repair. *Journal of Endovascular Therapy*.

[34] Murray D, Ghosh J, Khwaja N, Murphy MO, Baguneid MS, Walker MG (2006). Access for endovascular aneurysm repair. *Journal of Endovascular Therapy*.

[35] Kritpracha B, Pigott JP, Russell TE (2002). Bell-bottom aortoiliac endografts: an alternative that preserves pelvic blood flow. *Journal of Vascular Surgery*.

[36] McDonnell CO, Semmens JB, Allen YB, Jansen SJ, Brooks DM, Lawrence-Brown MMD (2007). Large iliac arteries: a high-risk group for endovascular aortic aneurysm repair. *Journal of Endovascular Therapy*.

[37] Frigatti P, Lepidi S, Piazza M (2010). A new endovascular approach to exclude isolated bilateral common iliac artery aneurysms. *European Journal of Vascular & Endovascular Surgery*.

[38] Parsons RE, Marin ML, Veith FJ, Parsons RB, Hollier LH (1999). Midterm results of endovascular stented grafts for the treatment of isolated iliac artery aneurysms. *Journal of Vascular Surgery*.

[39] Boules TN, Selzer F, Stanziale SF (2006). Endovascular management of isolated iliac artery aneurysms. *Journal of Vascular Surgery*.

[40] Greenberg RK, West K, Pfaff K (2006). Beyond the aortic bifurcation: branched endovascular grafts for thoracoabdominal and aortoiliac aneurysms. *Journal of Vascular Surgery*.

[41] Karthikesalingam A, Hinchliffe RJ, Holt PJE, Boyle JR, Loftus IM, Thompson MM (2010). Endovascular aneurysm repair with preservation of the internal iliac artery using the iliac branch graft device. *European Journal of Vascular and Endovascular Surgery*.

[42] Naik J, Hayes PD, Sadat U, See TC, Cousins C, Boyle JR (2008). Internal iliac artery branch graft for common iliac artery aneurysm following previous open abdominal aortic aneurysm repair. *European Journal of Vascular and Endovascular Surgery*.

